# Comparative pathogenesis of COVID-19, MERS, and SARS in a nonhuman primate model

**DOI:** 10.1126/science.abb7314

**Published:** 2020-04-17

**Authors:** Barry Rockx, Thijs Kuiken, Sander Herfst, Theo Bestebroer, Mart M. Lamers, Bas B. Oude Munnink, Dennis de Meulder, Geert van Amerongen, Judith van den Brand, Nisreen M. A. Okba, Debby Schipper, Peter van Run, Lonneke Leijten, Reina Sikkema, Ernst Verschoor, Babs Verstrepen, Willy Bogers, Jan Langermans, Christian Drosten, Martje Fentener van Vlissingen, Ron Fouchier, Rik de Swart, Marion Koopmans, Bart L. Haagmans

**Affiliations:** 1 Department of Viroscience, Erasmus University Medical Center, Rotterdam, Netherlands.; 2 Viroclinics Xplore, Schaijk, Netherlands.; 3 Department of Virology, Biomedical Primate Research Centre, Rijswijk, Netherlands.; 4 Animal Science Department, Biomedical Primate Research Centre, Rijswijk, Netherlands.; 5 Population Health Sciences, Unit Animals in Science and Society, Faculty of Veterinary Medicine, Utrecht University, Netherlands.; 6 Institute of Virology, Charité-Universitätsmedizin, Berlin, Germany.; 7 Erasmus Laboratory Animal Science Center, Erasmus University Medical Center, Rotterdam, Netherlands.

## Abstract

The current pandemic coronavirus, severe acute respiratory syndrome–coronavirus 2 (SARS-CoV-2), was recently identified in patients with an acute respiratory syndrome, coronavirus disease 2019 (COVID-19). To compare its pathogenesis with that of previously emerging coronaviruses, we inoculated cynomolgus macaques with SARS-CoV-2 or Middle East respiratory syndrome (MERS)–CoV and compared the pathology and virology with historical reports of SARS-CoV infections. In SARS-CoV-2–infected macaques, virus was excreted from nose and throat in the absence of clinical signs and detected in type I and II pneumocytes in foci of diffuse alveolar damage and in ciliated epithelial cells of nasal, bronchial, and bronchiolar mucosae. In SARS-CoV infection, lung lesions were typically more severe, whereas they were milder in MERS-CoV infection, where virus was detected mainly in type II pneumocytes. These data show that SARS-CoV-2 causes COVID-19–like disease in macaques and provides a new model to test preventive and therapeutic strategies.

## Coronavirus in nonhuman primates

We urgently need vaccines and drug treatments for coronavirus disease 2019 (COVID-19). Even under these extreme circumstances, we must have animal models for rigorous testing of new strategies. Rockx *et al.* have undertaken a comparative study of three human coronaviruses in cynomolgus macaques: severe acute respiratory syndrome–coronavirus (SARS-CoV) (2002), Middle East respiratory syndrome (MERS)–CoV (2012), and SARS-CoV-2 (2019), which causes COVID-19 (see the Perspective by Lakdawala and Menachery). The most recent coronavirus has a distinct tropism for the nasal mucosa but is also found in the intestinal tract. Although none of the older macaques showed the severe symptoms that humans do, the lung pathology observed was similar. Like humans, the animals shed virus for prolonged periods from their upper respiratory tracts, and like influenza but unlike the 2002 SARS-CoV, this shedding peaked early in infection. It is this cryptic virus shedding that makes case detection difficult and can jeopardize the effectiveness of isolation.

*Science*, this issue p. 1012; see also p. 942

## Teaser

SARS-CoV-2 infection in macaques results in COVID-19–like disease with prolonged virus shedding from the nose and throat in the absence of symptoms.

After the first reports of an outbreak of an acute respiratory syndrome in China in December 2019, a novel coronavirus, severe acute respiratory syndrome–coronavirus 2 (SARS-CoV-2), was identified (*1*, *2*). As of 14 March 2020, over 140,000 cases were reported worldwide with more than 5400 deaths, surpassing the combined number of cases and deaths of two previously emerging coronaviruses, SARS-CoV and Middle East respiratory syndrome (MERS)–CoV (*3*). The disease caused by this virus, coronavirus disease 2019 (COVID-19), is characterized by a range of symptoms, including fever, cough, dyspnoea, and myalgia in most cases (*2*). In severe cases, bilateral lung involvement with ground-glass opacity is the most common chest computed tomography (CT) finding (*4*). Similarly to the 2002–2003 outbreak of SARS, the severity of COVID-19 disease is associated with increased age and/or a comorbidity, although severe disease is not limited to these risk groups (*5*). However, despite the large number of cases and deaths, limited information is available on the pathogenesis of this virus infection. Two reports on the histological examination of the lungs of three patients showed bilateral diffuse alveolar damage (DAD), pulmonary edema, and hyaline membrane formation, indicative of acute respiratory distress syndrome (ARDS), as well as characteristic syncytial cells in the alveolar lumen (*6*, *7*), similar to findings during the 2002–2003 outbreak of SARS-CoV (*8*). The pathogenesis of SARS-CoV infection was previously studied in a nonhuman primate model (cynomolgus macaques) where aged animals were more likely to develop disease (*9*–*13*). In the current study, SARS-CoV-2 infection was characterized in the same animal model and compared with infection with MERS-CoV and historical data on SARS-CoV (*9*, *10*, *12*).

First, two groups of four cynomolgus macaques [both young adult (young), 4 to 5 years of age; and old adult (aged), 15 to 20 years of age] were inoculated by a combined intratracheal (IT) and intranasal (IN) route with a SARS-CoV-2 strain from a German traveler returning from China. No overt clinical signs were observed in any of the infected animals, except for a serous nasal discharge in one aged animal on day 14 post inoculation (p.i.). No significant weight loss was observed in any of the animals during the study. By day 14 p.i., all remaining animals seroconverted as revealed by the presence of SARS-CoV-2–specific antibodies against the virus S1 domain and nucleocapsid proteins in their sera (fig. S1).

**Fig. 1:**
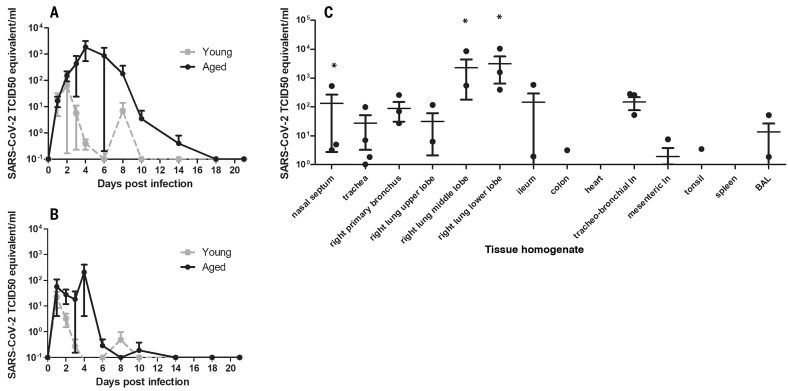
Virus shedding and virus detection in organs of SARS-CoV-2–inoculated cynomolgus macaques. Viral RNA was detected in nasal (**A**) and throat (**B**) swabs and in tissues (**C**) of SARS-CoV-2–infected animals by RT-qPCR. Samples from four animals (days 1 to 4) or two animals (days >4) per group were tested. The error bars represent the SEM. Virus was detected in tissues from two young and two aged animals on day 4 by RT-qPCR. Asterisk (*) indicates that infectious virus was isolated.

As a measure of virus shedding, nasal, throat, and rectal swabs were assayed for virus by reverse transcription–quantitative polymerase chain reaction (RT-qPCR) and virus culture. In nasal swabs, SARS-CoV-2 RNA peaked by day 2 p.i. in young animals and by day 4 p.i. in aged animals and was detected up to at least day 8 p.i. in two out of four animals and up to day 21 p.i. in one out of four animals (Fig. 1A). Overall, higher levels of SARS-CoV-2 RNA were detected in nasal swabs of aged animals compared with young animals. SARS-CoV-2 RNA in throat swabs peaked at day 1 p.i. in young and day 4 p.i. in aged animals and decreased more rapidly over time by comparison with the nasal swabs but could still be detected intermittently up to day 10 p.i. (Fig. 1B). Low levels of infectious virus were cultured from throat and nasal swabs up to day 2 and 4, respectively (table S1). In support of virus shedding by these animals, environmental sampling was performed to determine potential contamination of surfaces. Environmental sampling indicated the presence of low levels of SARS-CoV-2 RNA on surfaces through both direct contact (hands) and indirect contamination within the isolator (table S2). SARS-CoV-2 RNA was only detected in a rectal swab from one animal on day 14 p.i., and no viral RNA was detected in whole blood at any time point throughout the study.

**Fig. 2:**
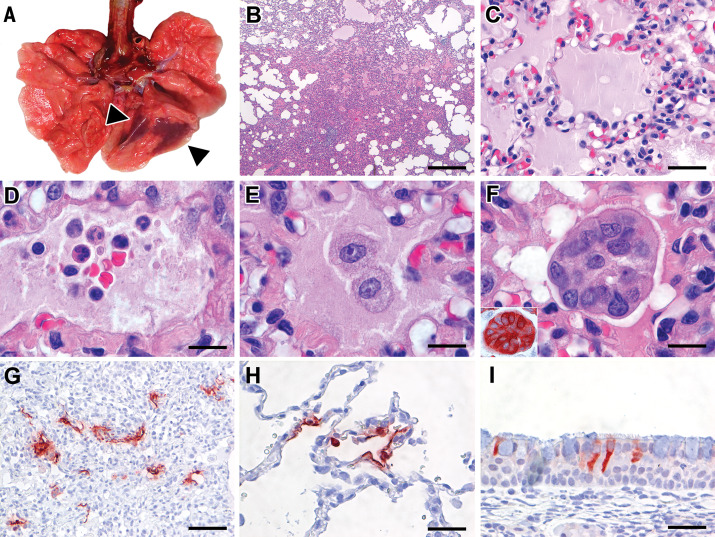
Characteristic pathological changes and virus antigen expression in the lungs of SARS-CoV-2–inoculated cynomolgus macaques. (**A**) Two foci of pulmonary consolidation in the left lower lung lobe (arrowheads). (**B**) Area of pneumonia [staining with hematoxylin and eosin (H&E); bar, 0.5 cm). (**C**) Edema fluid in alveolar lumina (H&E; bar, 25 μm). (**D**) Neutrophils, as well as erythrocytes, fibrin, and cell debris, in an alveolar lumen flooded by edema fluid (H&E; bar, 10 μm). (**E**) Mononuclear cells, either type II pneumocytes or alveolar macrophages, in an alveolar lumen flooded by edema fluid (H&E; bar, 10 μm). (**F**) Syncytium in an alveolar lumen (H&E; 100× objective). Inset: Syncytium expresses keratin, indicating epithelial cell origin [immunohistochemistry (IHC) for pankeratin AE1/AE3; bar, 10 μm]. (**G**) SARS-CoV-2 antigen expression is colocalized with areas of diffuse alveolar damage (IHC for SARS-CoV-nucleocapsid; bar, 50 μm). (**H**) Type I (flat) and type II (cuboidal) pneumocytes in affected lung tissue express SARS-CoV-2 antigen (IHC for SARS-CoV-nucleocapsid; bar, 25 μm). (**I**) Ciliated columnar epithelial cells of respiratory mucosa in nasal cavity express SARS-CoV-2 antigen (IHC for SARS-CoV-nucleocapsid; bar, 25 μm).

On autopsy of four macaques on day 4 p.i., two had foci of pulmonary consolidation in the lungs (Fig. 2A). One animal (aged: 17 years) showed consolidation in the right middle lobe, representing less than 5% of the lung tissue. A second animal (young: 5 years) had two foci in the left lower lobe, representing about 10% of the lung tissue (Fig. 2A). The consolidated lung tissue was well circumscribed, red-purple, level, and less buoyant than normal. The other organs in these two macaques, as well as the respiratory tract and other organs of the other two animals, were normal.

Virus replication was assessed by RT-qPCR on day 4 p.i. in tissues from the respiratory, digestive, urinary, and cardiovascular tracts and from endocrine and central nervous systems, as well as from various lymphoid tissues. Virus replication was primarily restricted to the respiratory tract (nasal cavity, trachea, bronchi, and lung lobes) with highest levels of SARS-CoV-2 RNA in lungs (Fig. 1C). In three out of four animals, SARS-CoV-2 RNA was also detected in ileum and tracheo-bronchial lymph nodes (Fig. 1C).

The main histological lesion in the consolidated pulmonary tissues of both the young and aged animals involved the alveoli and bronchioles and consisted of areas with acute or more advanced DAD (Fig. 2B). In these areas, the lumina of alveoli and bronchioles were variably filled with protein-rich edema fluid, fibrin, and cellular debris; alveolar macrophages; and fewer neutrophils and lymphocytes (Fig. 2, C to E). There was epithelial necrosis with extensive loss of epithelium from alveolar and bronchiolar walls. Hyaline membranes were present in a few damaged alveoli. In areas with more advanced lesions, the alveolar walls were moderately thickened and lined by cuboidal epithelial cells (type II pneumocyte hyperplasia), and the alveolar lumina were empty. Alveolar and bronchiolar walls were thickened by edema fluid, mononuclear cells, and neutrophils. There were aggregates of lymphocytes around small pulmonary vessels. Moderate numbers of lymphocytes and macrophages were present in the lamina propria and submucosa of the bronchial walls, and a few neutrophils were detected in the bronchial epithelium. Regeneration of epithelium was seen in some bronchioles, visible as an irregular layer of squamous to high cuboidal epithelial cells with hyperchromatic nuclei. There were occasional multinucleated giant cells (syncytia) free in the lumina of bronchioles and alveoli (Fig. 2F) and, based on positive pan-keratin staining and negative CD68 staining, these appeared to originate from epithelial cells (Fig. 2F, inset).

SARS-CoV-2 antigen expression was detected in moderate numbers of type I pneumocytes and a few type II pneumocytes within foci of DAD (Fig. 2, G and H, and fig. S2). The pattern of staining was similar to that in lung tissue from SARS-CoV–infected macaques (positive control). SARS-CoV-2 antigen expression was not observed in any of the syncytia. In addition, SARS-CoV-2 antigen expression was detected in nonlesional tissues of all lung lobes in three out of four macaques (both young and one aged) in a few type I and II pneumocytes, bronchial ciliated epithelial cells, and bronchiolar ciliated epithelial cells. The other aged macaque, without virological or pathological evidence of SARS-CoV-2 infection in the lungs, did have SARS-CoV-2 antigen expression in ciliated epithelial cells of nasal septum (Fig. 2I), nasal concha, and palatum molle, in the absence of associated histopathological changes. No SARS-CoV antigen expression was detected in other sampled tissues, including brain and intestine.

To assess the severity of infection with SARS-CoV-2 compared with MERS-CoV, we inoculated young cynomolgus macaques (3 to 5 years of age) with MERS-CoV via the IN and IT route. All animals remained free of clinical signs. At day 21 p.i., all remaining animals (*n* = 2) seroconverted as revealed by the presence of MERS-CoV–specific antibodies in their sera by ELISA (fig. S3).

**Fig. 3:**
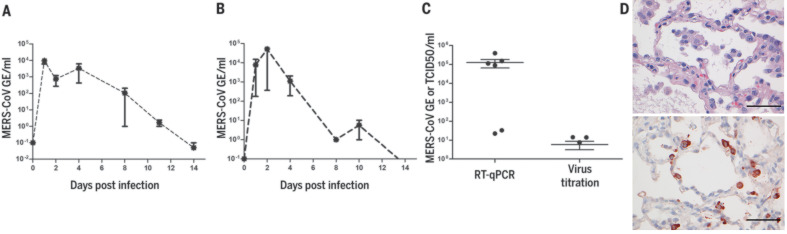
Virus shedding and virus detection in organs of MERS-CoV–inoculated cynomolgus macaques. Viral RNA was detected in nasal (**A**) and throat (**B**) swabs and tissues (**C**) of MERS-CoV–infected animals by RT-qPCR. Samples from four animals per group were tested. The error bars represent the SEM. Virus was detected in tissues on day 4 by RT-qPCR. Histopathological changes (**D**) (left) with hypertrophic and hyperplastic type II pneumocytes in the alveolar septa and increased numbers of alveolar macrophages in the alveolar lumina and virus antigen expression (right) in type II pneumocytes. Bar, 50 μm.

MERS-CoV RNA was detected in nasal (Fig. 3A) and throat swabs (Fig. 3B) on days 1 to 11 p.i., with peaks on days 1 and 2 p.i., respectively. Low levels [between 1 and 85 median tissue culture infectious dose (TCID50) equivalent/ml] of MERS-CoV RNA were detected in rectal swabs on days 2 and 3 p.i.

At autopsy of four macaques at day 4 p.i., three animals had foci of pulmonary consolidation, characterized by slightly depressed areas in the lungs, representing less than 5% of the lung tissue (Table 1). Similar to SARS-CoV-2 infection in both young and aged animals, on day 4 p.i., MERS-CoV RNA was primarily detected in the respiratory tract of inoculated animals (Fig. 3C). Infectious virus titers were comparable to those of SARS-CoV-2 infection, but lower compared to SARS-CoV infection, of young macaques (Table 1). In addition, MERS-CoV RNA was detected in the spleen (Table 1).

**Table 1:** Comparative pathogenesis of SARS-CoV-2, MERS-CoV, and SARS-CoV infections in cynomolgus macaques. Max, maximum; Ref., reference.

**Virus**	**Age** **category**	**No.**	**Clinical** **signs**	**Max.** **excre** **tion from** throat*	**Max.** **excre** **tion from** nose*	**Viral titer** **in lung†**	**Virus in** **extra-** **respir** **atory** **tissues**	**Pulmonary lesions at 4 days post inoculation**					**Ref.**
								**% affect** **ed lung**	**Hyaline** **mem** **branes**	**Alveolar** **edema**	**Leuko** **cyte** **infiltra** **tion**	**Cell type** **tropism**	
**SARS-** **CoV**	Young	4	No	10e3.5	10e4.2	10e6.5	No	0–5	No	No	Yes	Type I & II pneumo cytes	(*10*–*12*)
**SARS-** **CoV**	Aged	4	Yes	10e3.0	10e3.0	10e6.2	Kidney	0–60	Yes	Yes	Yes	Type I & II pneumo cytes	(*10*–*12*)
**MERS-** **CoV**	Young	4	No	10e5.3	10e4.3	10e4.4	Spleen	0–5	No	Yes (small amount)	Yes	Type II pneumo cytes	This study
**SARS-** **CoV-2**	Young	2	No	10e1.8	10e2.4	10e4.0	Ileum, colon, tonsil	0–10	Yes	Yes	Yes	Type I & II pneumo cytes	This study
**SARS-** **CoV-2**	Aged	2	No	10e2.9	10e3.7	10e3.1	Ileum, colon, tonsil	0–5	No	No	Yes	Type I & II pneumo cytes	This study

*TCID50eq per ml.

†TCID50eq per gram of tissue.

Consistent with the presence of virus in the lower respiratory tract at day 4 p.i., histopathological changes characteristic for DAD were observed in the lungs of inoculated animals (Fig. 3D). The alveolar septa were thickened owing to infiltration of neutrophils and macrophages, and to moderate type II pneumocyte hyperplasia and hypertrophy. In the alveolar lumina, there were increased numbers of alveolar macrophages and some edema fluid containing fibrin and some neutrophils (Fig. 3D). Few syncytial cells were seen in the alveolar lumina. MERS-CoV antigen was not detected in tissues on day 4 p.i. in any part of the respiratory tract. We therefore sampled four young macaques at day 1 p.i. At this time, we observed multifocal expression of viral antigen, predominantly in type II pneumocytes and occasionally in type I pneumocytes, bronchiolar and bronchial epithelial cells, and some macrophages (Fig. 3D).

In summary, we inoculated young and aged cynomolgus macaques with a low-passage clinical isolate of SARS-CoV-2, which resulted in productive infection in the absence of overt clinical signs. Recent studies in human cases have shown that presymptomatic and asymptomatic cases can also shed virus (*14*, *15*). Increased age did not affect disease outcome, but there was prolonged viral shedding in the upper respiratory tract of aged animals. Prolonged shedding has been observed in both SARS-CoV-2 and SARS-CoV patients (*16*, *17*). SARS-CoV-2 shedding in our asymptomatic model peaked early in the course of infection, similar to what is seen in symptomatic patients (*16*). Also, SARS-CoV-2 antigen was detected in ciliated epithelial cells of nasal mucosae at day 4 p.i., which was not seen for SARS-CoV (*10*) or MERS-CoV infections (this study) in this animal model. Viral tropism for the nasal mucosa fits with efficient respiratory transmission, as has been seen for influenza A virus (*18*). This early peak in virus shedding for SARS-CoV-2 is similar to that of influenza virus shedding (*19*) and may explain why case detection and isolation may not be as effective for SARS-CoV-2 as it was for the control of SARS-CoV (*20*). SARS-CoV-2 was primarily detected in tissues of the respiratory tract; however, SARS-CoV-2 RNA was also detectable in other tissues such as intestines, in line with a recent report (*21*). Similar results regarding viral shedding and tissue and cell tropism were recently also reported after SARS-CoV-2 inoculation in rhesus macaques. However, unlike in our model, SARS-CoV-2 infection in rhesus macaques does result in transient respiratory disease and weight loss (*22*, *23*).

Two out of four animals had foci of DAD on day 4 p.i. The colocalization of SARS-CoV-2 antigen expression and DAD provides strong evidence that SARS-CoV-2 infection caused this lesion. The histological character of the DAD, including alveolar and bronchiolar epithelial necrosis, alveolar edema, hyaline membrane formation, and accumulation of neutrophils, macrophages, and lymphocytes, corresponds with the limited pathological analyses of human COVID-19 cases (*6*, *7*). In particular, the presence of syncytia in the lung lesions is characteristic of respiratory coronavirus infections. Whereas MERS-CoV primarily infects type II pneumocytes in cynomolgus macaques, both SARS-CoV and SARS-CoV-2 also infect type I pneumocytes. Injury to type I pneumocytes can result in pulmonary edema, and formation of hyaline membranes (*24*), which may explain why hyaline membrane formation is a hallmark of SARS and COVID-19 (*7*, *10*) but not frequently reported for MERS (*25*, *26*).

These data show that cynomolgus macaques are permissive to SARS-CoV-2 infection, shed virus for a prolonged period of time, and display COVID-19–like disease. In this nonhuman primate model, SARS-CoV-2 replicates efficiently in respiratory epithelial cells throughout the respiratory tract, including nasal cavity, bronchi, bronchioles, and alveoli. Replication in the upper respiratory tract fits with efficient transmission between hosts, whereas replication in the lower respiratory tract fits with the development of lung disease. An in-depth comparison of infection with SARS-CoV, MERS-CoV, and SARS-CoV-2 in this model may identify key pathways in the pathogenesis of these emerging viruses. This study provides a new infection model that will be critical in the evaluation and licensure of preventive and therapeutic strategies against SARS-CoV-2 infection for use in humans, as well as for evaluating the efficacy of repurposing species-specific existing treatments, such as pegylated interferon (*12*).

## Supplementary Materials

science.sciencemag.org/content/368/6494/1012/suppl/DC1 Materials and Methods Figs. S1 to S3 Tables S1 and S2 References (*27*–*30*) MDAR Reproducibility Checklist
